# Use of Simulated Patients to Evaluate Combined Oral Contraceptive Dispensing Practices of Community Pharmacists

**DOI:** 10.1371/journal.pone.0079875

**Published:** 2013-12-04

**Authors:** Paulo Roque Obreli-Neto, Leonardo Régis Leira Pereira, Camilo Molino Guidoni, André de Oliveira Baldoni, Srecko Marusic, Divaldo Pereira de Lyra-Júnior, Kelsen Luis de Almeida, Ana Claudia Montolezi Pazete, Janaina Dutra do Nascimento, Mitja Kos, Edmarlon Girotto, Roberto Kenji Nakamura Cuman

**Affiliations:** 1 Department of Pharmacology and Therapeutics, State University of Maringa, Maringa, Brazil; 2 Department of Pharmaceutical Sciences, Faculty of Pharmaceutical Sciences of Ribeirao Preto, University of Sao Paulo, Ribeirao Preto, Sao Paulo, Brazil; 3 Department of Pharmaceutical Sciences, State University of Londrina, Londrina, Parana, Brazil; 4 Department of Clinical Pharmacology, University Hospital Dubrava, Zagreb, Croatia; 5 Laboratory of Teaching and Research in Social Pharmacy, Federal University of Sergipe, São Cristóvão, Sergipe, Brazil; 6 Department of Pharmacy, Faculdades Integradas de Ourinhos, Sao Paulo, Brazil; 7 Chair of Social Pharmacy, University of Ljubljana, Faculty of Pharmacy, Ljubljana, Slovenia; Groningen Research Institute of Pharmacy, Netherlands

## Abstract

**Background:**

Combined oral contraceptive (COC) use is the most commonly used reversible method of birth control. The incorrect use of COCs is frequent and one of the most common causes of unintended pregnancies. Community pharmacists (CPs) are in a strategic position to improve COC use because they are the last health professional to interact with patients before drug use.

**Objective:**

To evaluate the COC dispensing practices of CPs in a developing country.

**Method:**

A cross-sectional study was conducted in community pharmacies of Assis and Ourinhos microregions, Brazil, between June 1, 2012, and October 30, 2012. Four simulated patients (SPs) (with counseled audio recording) visited community pharmacies with a prescription for Ciclo 21^®^ (a COC containing ethinyl estradiol 30 mcg + levonorgestrel 15 mcg). The audio recording of every SP visit was listened to independently by 3 researchers to evaluate the COC dispensing practice. The percentage of CPs who performed a screening for safe use of COCs (i.e., taking of patients’ medical and family history, and measuring of blood pressure) and provided counseling, as well as the quality of the screening and counseling, were evaluated.

**Results:**

Of the 185 CPs contacted, 41 (22.2%) agreed to participate in the study and finished the study protocol. Only 3 CPs asked the SP a question (1 question asked by each professional), and all of the questions were closed-ended, viz., “do you smoke?” (n = 2) and “what is your age?” (n = 1). None of the CPs measured the patient’s blood pressure. Six CPs provided counseling when dispensing COCs (drug dosing, 5 CPs; possible adverse effects, 2 CPs), and one CP provided counseling regarding both aspects.

**Conclusion:**

The CPs evaluated did not dispense COC appropriately and could influence in the occurrence of negatives therapeutic outcomes such as adverse effects and treatment failure.

## Introduction

The use of combined oral contraceptives (COC) is the most common reversible method of birth control in developed and developing countries. Nearly 100 million women worldwide use this method. COCs contain a combination of synthetic estrogen and a steroid with progestational activity; appropriate use of COCs can yield contraceptive efficacy greater than 99% [1]. Besides prevention of pregnancy, there are several other non-contraceptive benefits associated with the use of COC, such as reduction in the risk of ovarian and endometrial cancers and improved regulation of menstruation [2,3].

Despite the contraceptive and non-contraceptive benefits, COCs are associated with a high self-reported 12-month discontinuation rate (approximately 50%). COC discontinuation has been reported to lead to unintended pregnancies in more than 50% of cases [1,4]. Unintended pregnancies carry a huge humanistic and economic burden to the individuals and to society, with an estimated cost of $11.1 billion each year in the United States [5]. Access to COCs in a majority of countries is by prescription only, making it necessary to contact a physician to have access to these pills. This form of access poses some difficulties for many women who wish to acquire COCs, and is reported as one of the most common reasons for not using COCs, or introducing gaps in the use of COCs [4,6,7]. Other frequently reported barriers to COC adherence is a lack of patients’ knowledge of COC contraceptive and non-contraceptive benefits, suggesting that health care professionals need to provide adequate evidence-based contraceptive counseling [4,8]. These results suggest a need for developing practices that improve long-term COC adherence.

Currently, there is a debate about whether over-the-counter (OTC) access to COCs should be allowed, with an attempt to improve COC access and use [9,10]. However, no drug or intervention is completely without risk of adverse effects for the patient. Women presenting with specific pre-existing conditions are at an increased risk of developing myocardial infarction, stroke, thromboembolism (DVT)/pulmonary embolism (PE), and other health problems associated with the use of COCs [11,12]. These pre-existing conditions included age > 35 years, smoking of >15 cigarettes/day, elevated blood pressure levels (systolic > 160 mmHg or diastolic > 100 mmHg), diabetes with nephropathy/retinopathy/neuropathy or other vascular disease, diabetes a duration of >20 years, migraine with aura, presence or history of DVT or PE, history of ischemic heart disease, history of cerebrovascular accident, complicated valvular heart disease, and less than <21 days after childbirth (with or without other risk factors for venous thromboembolism). It is recommended that health professionals conduct a screening for safe use before the patient begins using COCs. This screening should involve evaluation of the patient’s medical history, family history and blood pressure before the patient begins using COCs, with periodic evaluation thereafter [13,14].

Previous pharmacoepidemiological studies have reinforced the importance of conducting a screening to insure the safe use of COCs, given the significant number of women in whom these agents are contraindicated. For example, one study using data from the National Health and Nutrition Examination Survey (NHANES) found that approximately 16% of women of reproductive age in the U.S. had at least one contraindication for COC use [15]. A study carried out in the U.S. city of El Paso found that approximately 39% of women of reproductive age had at least one contraindication for COC, noting that migraine with aura (17%) and hypertension (blood pressure of 140/90 mmHg or higher; 14%) were amongst the most common contraindications [16]. Of the conditions in which COCs were contraindicated in an estimated 18% women of reproductive age in areas along the U.S.-Mexico border, hypertension (8%) was the most common condition, followed by smoking at age 35 or older (5%), and migraine with aura (4%) [17].

The results of previous studies [16,18] verified that women could perform self-screening for contraindications as well as physicians, but some practical considerations must be addressed in reference to these studies. First, women in these studies reviewed COC contraindications by using a checklist developed by specialists, an option not offered at practitioner’s offices and an activity that would be difficult to implement on a large scale in clinical practice. This is particularly difficult to implement in developing countries with few economic resources invested in health policies and high rates of health illiteracy. Second, the patients recruited for these studies had a higher mean level of education, which did not represent the educational profile of women in many developing countries and can have direct effects on the self-screening process. For example, the findings of a study carried out in areas along the U.S.-Mexico border supported the hypothesis that women who obtain COCs OTC in a real-life situation are more likely to experience adverse effects than patients who obtain COCs with a medical prescription [17]. These results highlight the need to adopt strategies that are better adapted to the reality of developing countries, and the real-life situations of women in these countries.

Community pharmacists (CPs) could play a vital role in the access to COCs (OTC or prescription), since they are in a strategic position to help screen patients for contraindications and counsel patients during the dispensing process on the correct use of COCs. Prior to drug consumption, the process of drug dispensation (either OTC or prescription) is the patient’s last point of contact with a health professional, an action performed and/or supervised by a CP in the majority of countries. Pharmacy-based screening for safe use of COCs, and counseling regarding the correct use, benefits, and adverse effects of COCs can improve patient adherence and safety [19]. Based on the results of previous studies indicating the potential and ability of CPs to provide effective clinical services in the community setting, the American College of Clinical Pharmacy (ACCP) supports the change of COCs to OTC with the condition that they are sold where a pharmacist is on duty [20]. The ACCP also states that there is a need to evaluate how COCs are actually dispensed, in order to identify the CPs’ role in COC access [20]. However, to the best of our knowledge, there are few studies evaluating the COC dispensing practices of CPs either OTC or with a prescription.

Although many women use COCs, very little is known about COC dispensing practices compared to what is known about the dispensing practice of emergency contraception pills (ECPs) [21-24]. Previous studies verified that the practice of dispensing ECPs is not carefully undertaken, with infrequent collection of patient history and limited engagement in patient counseling [21-24]. Differences in indication, drug dosage, and contraindications comparing ECPs and COCs also make it difficult to extrapolate predictions for COCs from published data.

Additionally, most of the earlier studies administered self-reported questionnaires to CPs, or conducted patient interviews to evaluate dispensing practices [25-27], which could have led to an overestimation of the rate of effective COC dispensing practices. For example, in methods such as self-reported questionnaires and interviews, the CPs and/or the patients know that a test is being conducted. This can alter the testee’s performance, yielding results that do not express what actually happens in the pharmacy practice setting [28]. In this respect, the simulated patient (SP) method has been shown to help overcome the methodological problems of other quantitative methods like self-reported questionnaires and interviews, and has become a useful and objective tool for evaluating professional performance [29]. The SP method focuses on actual behaviors rather than proxy measures [30]. A SP is an individual who is trained to visit a pharmacy and enact a scenario previously developed by researchers that tests a specific behavior of the pharmacist or pharmacy staff member. The professionals who are being audited are unaware that the SP is not a genuine patient and that they are being evaluated [30]. The aim of this study was to evaluate the COC dispensing practice of CPs by using the SP method.

## Methods

### Study design and setting

This study was approved by the Research Ethics Committee of the State University of Maringa, Brazil. COC dispensing practices of CPs were evaluated using the SP method. A cross-sectional study was conducted from June 1, 2012 to October 30, 2012 in community pharmacies of the Assis and Ourinhos microregions in Brazil. The Assis and Ourinhos microregions cover 29 municipalities and have an estimated population of 544,000 that is served by a total of 185 community pharmacies. Brazilian community pharmacies are private health establishments where drugs, medical products, and other products such as toiletries and beauty products are dispensed, either with or without a prescription (depending on the specific legislation for the product).

In Brazilian community pharmacies, legislation necessitates the presence of at least one pharmacist who is responsible for the community pharmacy at all times. Establishments must also list on its employee roster the number of pharmacists required to conform to this legislation. To assume the responsibility of a community pharmacy, an individual must have a degree in pharmacy from an institution recognized by the Brazilian Ministry of Education and must be registered with the Regional Board of Pharmacy of the state of practice [31].

In Brazil, is estimated that 27.4% of women aged 15–44 years use COCs [32]. The access to COCs in Brazil is by prescription only [33]. Brazilian sanitary legislation on drug dispensing establishes that (1) CPs should evaluate medical prescriptions for the presence of signs of tampering and/or erasures, legibility, patient identification, drug identification, drug dosage, pharmaceutical specialty, posology, duration of treatment, prescriber identification, and date/place of prescription, and that (2) CPs must guarantee to the patient the right of access to information and counseling regarding the correct use of drugs [34]. With respect to patient counseling, compliance with prescribed drug dosage, influence of foods on drug therapy, potential adverse drug reactions, and drug storage conditions are considered important topics by the Brazilian sanitary legislation [34]. However, there are no legal instruments to evaluate whether the Brazilian CPs are fulfilling the requirements of this sanitary legislation on drug dispensation. Besides this, there are no protocols recommended by pharmaceutical authorities or other organizations in Brazil that mandate CPs to screen for the safe use of COCs, and the counseling of women regarding the correct use of COCs.

### Study population

CPs were eligible for inclusion in the study if during the study period they were employees of a participating community pharmacy registered with the Regional Board of Pharmacy of Sao Paulo State, and worked during commercial hours (Monday to Friday, 8 am to 5 pm). Exclusion criteria included employment in 2 or more participating community pharmacies (to avoid evaluating the same CP more than once).

Every community pharmacy in the above-mentioned microregions was visited by 8 researchers during commercial hours. These researchers were pharmacy school graduates, and members of our research team. They did not receive any compensation for participating in the study. The researchers contacted the CPs in each establishment to invite them to participate in the study. The researchers explained to the CPs that the objective of the study was to evaluate the practice of dispensing using the SP method. Information regarding the SP method and study protocol was also provided. Eligible CPs were informed that an SP would ask the pharmacist to dispense a single drug prescription between June and October 2012. The SP would buy the prescribed drug and leave the pharmacy without informing the CP that the purchase was simulated. The details of the scenarios and the identity of the SPs were not disclosed. Thus, the study was conducted covertly in order to obtain reliable results [29]. Participants were assured that the data gathered would be kept anonymous and strictly confidential. The employees could refuse to participate at any time. CPs that agreed to participate in the study were evaluated for eligibility criteria, and some of their general characteristics were recorded (age, sex, function in the pharmacy, duration of experience as a CP, and hours worked per week).

### Simulated Patients

Four female pharmacy students of a Brazilian University (Faculdades Integradas de Ourinhos - FIO) were selected to be the SPs. They were aged 20–25 years, and were enrolled in their third year of pharmacy graduate studies (having completed classes in anatomy, physiology, and pharmacology) when they were selected and when they carried out the visits. The selection process was based on academic grades in the courses mentioned above, with female students aged 20–25 years having the best mean grade point average (GPA) being invited to participate. Four female students with the highest GPAs were accepted to participate in the study. These students were not reimbursed for their participation. 

The selected students were trained and evaluated before their visits to the community pharmacies. Training lasted 10 hours and was conducted by a researcher who specialized in the SP method. Training was conducted in a classroom, and involved the following steps: explanation of the SP method, communication techniques, acting techniques, and the scene to be performed. To guarantee consistency in terms of the amount of information provided by the SPs in response to patients’ questions, the SPs were trained not to offer any information unless questioned and to refrain from asking the CP for any information.

After the end of the training, the students were evaluated. In the evaluation process, the SPs conducted a scenario in a model dispensary, with a doctoral pharmacy degree student acting as a pharmacist. This student was a part of our research team and had already participated in previous studies involving the SP method. An audio recording of this scenario was evaluated by the researcher specializing in the SP method in order to guarantee the quality and similarity of SP visits. Every SP evaluated was approved by the researcher to perform the SP visits. The SPs signed a contract with our research team in which they agreed to conform to the study protocols established. They also consented to the ethical code created for this study in order to maintain the anonymity of participating individuals and to protect the integrity of the data obtained.

### The scenario

A 21-year-old woman with a prescription for a COC (Ciclo 21^®^: ethinyl estradiol 30 mcg + levonorgestrel 15 mcg) visits a community pharmacy. She has never used any method of contraception. She has a stable relationship with her fiancé (her fiancé is the only sexual partner that she has had in her life). Her menstrual history is normal and her cycle is regular. She has epilepsy (she experienced her first seizure 10 years ago) and takes 200 mg oral carbamazepine tablets twice a day (since the first seizure). She has no other diagnosed diseases and does not use any other drug. Her family history does not present any contraindication for the use of COCs. She does not smoke. The prescription used in the scenario was obtained from a physician (gynecologist) who practices in the Ourinhos microregion. The physician was informed about the study and agreed to dispense 4 prescriptions for our research. The physician was not reimbursed for this.

This scenario involves a woman who presents with a condition in which the theoretical or proven risks usually outweigh the advantages of using COC [13]. Carbamazepine is an antiepileptic drug (AED) that may decrease the effectiveness of COCs and result in an unintended pregnancy [35,36]. Besides this, carbamazepine and some other AEDs present teratogenic effects [37,38]. The desirable outcome of this scenario was that the CP contacts the prescribing physician to inform him/her of the disease and drug used by the patient and discusses the implementation of safer strategies. We selected this scenario because of the high rate of concomitant AED and COC use in women of reproductive age [39,40].

### Study protocol

A researcher guided the SP to every participating community pharmacy 30–50 days after verbal and written consent was obtained from the CP. Each community pharmacy was visited once by only one of the 4 SPs. The researcher did not enter the community pharmacy because it was desirable for the SP to avoid being recognized by the CP.

The SP entered the pharmacy with an audio recorder enabled and asked for the CP. She asked the CP to dispense the drug prescribed in the medical prescription. After the dispensation, she bought the COC and left the pharmacy. The duration of each dispensation was determined. During the visits, the SPs also tried to identify the profession of the dispensing official by using either the bills received or identity badges present on the white coat of the CPs.

The audio recording from every participating community pharmacy was independently reviewed by 5 researchers who are specialists in the use of COCs. After listening to the audio recording, the researchers filled out a form specifically developed for this study to identify the frequency and quality of the screening and counseling provided by the CPs to the SPs. The form was developed by our research team, given the lack of clinical protocols developed by any pharmaceutical authorities/organizations as a screen for safe use and for counseling on the use of COCs. We developed this form based on the safety aspects identified in the World Health Organization’s Medical eligibility criteria for contraceptive use [13], and from guidelines for pharmacist-patient counseling recommended by a number of pharmaceutical authorities/ organizations [41-44] (Appendix S1 - Sample data collection sheet). This form was developed in an attempt to define a set of questions that should be posed to patients, as well as a list of counseling points that should be provided to the patient by the CPs during the dispensing of COCs. The forms filled out by the different researchers were checked, and items filled by 3 or more researchers were considered representative of screening and counseling. Two months after the study was completed, all audio recordings were erased.

Visits in which one of the following situations occurred were excluded from further evaluation:

- the CP detected the SP visit,
- the SP did not follow the scenario,
- the SP provided additional information that was not requested by the CP, and
- the audio recording was incomprehensible.

### Detection of the Simulated Patient Visits

It was important that the SPs were not detected by the participating CPs since the behavior of the CP could have been influenced if they suspected a covert visit. The researchers explained to the eligible CPs that the study had a formalized feedback system for detecting SP visits, in which the CPs would telephone the researchers when they suspected that an SP visit had occurred (the CPs were required to provide the date, time, and drug dispensed in the suspected visit). On the basis of the information provided, the researchers would be able to determine if a visit had been detected and eliminate it from further study.

### Data analysis

The following general CP characteristics were recorded: age, sex, function in the pharmacy, duration of experience as a CP, and hours worked per week. Descriptive statistics were used to analyze most variables. Results were presented as the median and corresponding interquartile intervals, mean ± standard deviation (SD), or as a proportion. Numerical variables were tested for normal distribution by using the Kolmogorov–Smirnov and Shapiro–Wilk tests. Analyses were performed using Statistica (StatSoft, Sao Caetano do Sul, SP, Brazil) version 8.0 software.

## Results

### General characteristics of the Community Pharmacists

Of the 185 CPs contacted, 41 (22.2%) agreed to participate, and completed the study protocol. None of the SP visits were excluded. None of the CPs called to inform the researchers of a suspected SP visit during the study. All SPs were attended by the CPs. The median duration of dispensation was 160 seconds (range: 109–174 seconds), and the lowest and highest dispensation times were 69 seconds and 204 seconds, respectively. Table 1 shows the general characteristics of the CPs. The CPs were young adults, with little more than 10 years of experience as CPs. More than 65% of these professionals did not have administrative functions and engaged in patient-focused activities during most of their work time (Table 1). The CPs’ dialogue with SPs was focused on providing information about the price of the COCs and the conditions of payment with discount.

**Table 1:** General characteristics of community pharmacists that participated in the study, June–October 2012 (n = 41).

**Characteristic**	**Value**
Median age, years (interquartile interval)	31 (28–39)
Female, n (%)	29 (70.7)
Function in the pharmacy, n(%)	
Employee of the pharmacy, without administrative functions	27 (65.9)
Employee of the pharmacy, with administrative functions (manager)	8 (19.5)
Owner of the pharmacy	6 (14.6)
Median experience as a community pharmacist, years (interquartile interval)	7 (5–11)
Median work time per week, h (interquartile interval)	44.0 (40.0–44.0)

### Screening for safe use of COCs

More than 90% of the CPs did not ask the SP any questions before dispensing the COC. Only 3 CPs asked the SPs questions (just one question asked per professional), and all of the questions were closed-ended. None of the CPs conducted a systematic simple medical history review, including a patient interview (consisting of open-ended and closed-ended questions) and physical examination (blood pressure measurement) before dispensing the COC to the SP. Every CP had their screening procedure for safe use of COCs rated as very poor by the evaluators (inter-rater agreement: kappa, 0.92; 95% confidence interval [CI], 0.84–0.99). Table 2 shows the questions asked by the CP prior to dispensing the COC.

**Table 2:** Questions asked by the community pharmacists before dispensing combined oral contraceptives, June–October, 2012.

**Question asked**	**n (%)**
Do you smoke?	2 (4.9)
What is your age?	1 (2.4)

### Counseling provided by the CPs during dispensation

In total, 35 CPs did not provide any counseling to the SP during dispensing. Six professionals provided counseling when dispensing COCs (Table 3). Regarding drug dose, each CP simply read the drug dose written in the drug prescription to the SP; no CP checked whether the SP understood what was counseled. The possible adverse effects indicated by the 2 CPs that provided counseling regarding side effects were headache, nausea, and weight gain. None of the CPs provided counseling regarding what the patient must do if any adverse effects occurred or measures to avoid or minimize adverse effects. A total of 6 CPs had their counseling process rated as poor by the evaluators (inter-rater agreement: kappa, 0.93; 95% CI, 0.84–0.99), and 35 CPs had their counseling process rated as very poor by the evaluators (inter-rater agreement: kappa, 0.91; 95% CI, 0.83–0.99).

**Table 3:** Counseling provided by the community pharmacists when dispensing combined oral contraceptives, June–October, 2012.

**Counseling**	**n (%)**
Drug dosing	5 (12.2)
Possible adverse effects	2 (4.9)

One pharmacist provided counseling regarding 2 aspects (drug dosing and possible adverse effects).

## Discussion

To our knowledge, this is the first study to use the SP method to evaluate the COC dispensing practices of CPs in a large number of cities in a developing country. Awareness of gaps in the COC dispensing practice could help in the development of interventions to address this public health problem. The results demonstrate that most CPs did not perform screening for the safe use of COCs (92.7%) neither did they provide counseling on COCs (85.4%). The professionals who attempted to perform screening and provide counseling did so inadequately. Efforts to improve the COC dispensing practice must be addressed in order to guarantee the rational use of COC as an OTC or as a prescription-only drug.

Previous studies indicated that women desire active participation of the CPs in the process of accessing COCs. In a study by Landau et al [45], women stated that they believe that CPs should provide information on instructional methods (75%), provide screening and counseling regarding the method (46%), and be available to answer questions (24%). In this study, women also reported that they were more comfortable assessing COCs as OTC if CPs participated in this process [45]. Almost every woman who participated in the Direct Access Study was satisfied or very satisfied with the CP services focused on the provision of COCs. They indicated a willingness to recommend this service to a friend, and were also willing to pay for this kind of service [19]. These results suggested that women wish to be screened for contraindications and counseled by CPs regarding the use of COC.

However, similar to the findings reported by previous studies, we found that many CPs did not ask the women any questions neither did they measure their blood pressure when dispensing COCs. In Mexico City, only 31% of pharmacy workers reported having asked women any questions before selling drugs to them [25]. Chirdan and Zoakah found that 32.7% of pharmacy and chemist shop workers self-indicated that did not ask any questions of women who bought oral contraceptives [26]. Ratanajamit and Chongsuvivatwong found that COCs were sold with little or no history-taking in the city of Hat Yai, Thailand [46]. In a study carried out in Jamaica evaluating OTC access to COCs, the majority of CPs denied COC access to SPs presenting contraindications to COC use and recommended that these women consult a doctor to find out whether the pill would be safe for them before attempting to use the method [47]. In this study the SPs gave appropriate cues of the presence of contraindications to the CPs (for example, the SPs informed the CPs that they were smokers and had hypertension) [47]. However, on a daily basis, how many women will understand the significance of and inform the CPs about the existence of possible COC contraindications? Therefore, we strongly support the approach whereby CPs must actively engage patients in order to identify these contraindications, even in situations where appropriate cues are provided, similar to the scenario of our study.

The lack of screening for the safe use of COCs by CPs could present some risks for women, because in some conditions, unacceptable health risks are associated with concurrent use of COCs, and in other conditions, the theoretical or proven risks usually outweigh the advantages of using a COC [13,14]. For example, one meta-analyses established that the relationship between COC users with hypertension and risk of acute myocardial infarction (AMI) was 2.48 times (95% CI, 1.91–3.22) higher than that among nonusers without hypertension [48]. The likelihood of developing an ischemic stroke in COC users with a history of migraine was approximately eightfold higher than that in women without migraine (relative risk, 8.72; 95% CI, 5.05–15.05) [49] The prevalence of such risk factors for COC use is significant in women of reproductive age (range, 16–39%) [15-17].

Taking patients’ medical and family history and measuring the patients’ blood pressure systematically (for example, when the patient starts using COCs and subsequently at specific time intervals such as 4 months) during dispensation could help identify the presence of these dangerous conditions. For example, in a systematic review, Curtis et al. verified that the odds ratios for AMI were generally higher among COC users who had not had their blood pressure checked (OR, 2.76–9.47; 95% CI, 1.36–24.1) than among COC users who had (OR, 1.07–3.48; 95% CI, 0.66–8.70) [50]. CPs are in a strategic position to provide these clinical services to women, and could reduce the occurrence of negative outcomes associated with the use of COCs. Future studies evaluating the effect of screening by CPs for safe use on the negative outcomes of COCs use are needed.

In our study, every question asked by the CPs was closed-ended. Closed-ended questions reduce the patient’s degree of openness (the patient will answer as either “yes” or “no,” or in a few words at the most) and cause the patient to become more passive during the interview process [51], which may result in the loss of important information. Open-ended questions are more difficult to formulate but allow patients to become more involved in the communication process by allowing them to present information in their own words. Open-ended questions are also more effective for gathering information [51,52]. CPs must be aware of the importance of asking open-ended questions when interacting with patients.

Few CPs provided counseling when dispensing COCs, and the counseling provided by these few CPs was inadequate. Ratanajamit and Chongsuvivatwong found that COCs were sold with little or no counseling in the city of Hat Yai, Thailand [46]. The counsel provided by Jamaicans CPs to COC users was also inadequate, with a negligible number of CPs providing counseling regarding the best time to take the pill, steps to be taken on missing a pill or pills, the appropriate time to use backup methods, and the adverse effects of COCs [47]. A study carried out in Kuwait revealed that few CPs counseled women regarding COC use, with 17.4% and 13.0% providing counseling on their use and their adverse effects, respectively [27]. Previous studies found that a lack of counseling is a leading cause for incorrect use of COCs (for example, discontinuation and wrong attitude when missing pills) [1,5,53]. It is known that thorough patient counseling by pharmacists may improve adherence to pharmacotherapy, clinical outcomes, and patient satisfaction with pharmacy care [54-56]. It is therefore important that CPs provide counseling while dispensing COCs, to improve the quality of COC use.

Several guidelines have been published by professional organizations regarding the content, conditions, and circumstances for patient counseling [41-44]. Most of these guidelines suggest that the following information may be provided: name and description of the drug, indications, route of administration, dose and dosage form, directions for use, duration of therapy, special directions, precautions, adverse effects, and contraindications. These guidelines state that pharmacists need to individually tailor their counseling based on the patients’ understanding, culture, feelings, and cognitive abilities [41-44]. However, in our study, the CPs only provided counseling regarding dosage and a few adverse effects. Furthermore, the CPs who were studied also did not try to individualize counseling according to patient needs. Efforts are needed to improve counseling in COC dispensing practice.

This lack of screening for safe use and counseling regarding COC use could be attributed to several factors. For example, one third of the CPs of our sample have administrative functions in community pharmacies, including activities related to the acquisition and inventory control of drugs, leading to limited time and manpower to perform clinical activity directed toward the patient. Another factor underlying this kind of behavior is the CP’s focus on the product instead of the patient. Thus, assessment of the patient’s information needs is based on their medicines instead of posing questions to the patients. Furthermore, CPs may assume that patients who request a specific medicine are already knowledgeable about its use, and that asking questions and giving additional advice would be patronizing and could lead to a negative patient reaction [57,58]. Additionally, while U.S. pharmacy graduate programs have curricular requirements encompassing the various aspects of hormonal contraceptive service provision, including screening, prescribing, and monitoring [20], Brazilian pharmacy graduate programs are deficient in this respect.

### Strengths and limitations

Our study used the SP methodology, which can be an effective method of deriving valid, reliable outcomes that are difficult to achieve by any other method in pharmacy practice research [59]. To guarantee quality and similarity across SP visits, the SPs who were recruited had a similar background and received formal training (consistent duration, content, and trainer, as described in the methodology section). They were also formally evaluated before the visits. Another strength of the study was the inclusion of a method to assess the face validity of SP visits, since the CPs’ behavior could have been influenced if a covert visit was suspected [60]. In our study, none of the SP visits were detected by the CPs, suggesting that our SPs were well trained and reliable. SP visits were audio-recorded to minimize potential biases associated with missing information (both omissions and distortions) that can occur when the content of an SP visit is self-reported by the SP following the visit to the pharmacy [61].

Despite these advantages, our study has some limitations. The Hawthorne effect could have occurred during SP visits, because the CPs were invited to participate in the study before the SP visits were conducted, a point that was necessary for meeting ethical standards. To minimize the Hawthorne effect, SP visits were conducted after a considerable interval (30–50 days) after the first contact with a CP. In addition, our study only involved CPs of 2 Brazilian microregions, and the generalization of our findings to other locations may be limited.

## Conclusion

Our results indicated that most CPs did not perform screening for safe use of COCs neither did they counsel the patients during drug dispensation. The few CPs that attempted to perform screening for safe use and counsel the patients, did so inadequately. These practice patterns may affect the occurrence of negative therapeutic outcomes such as adverse effects and treatment failure. Efforts are needed to improve COC dispensing practice in these Brazilian microregions to promote the rational use of COCs.

## Supporting Information

Appendix S1 **Sample data collection sheet.**
(DOC)
