# Supplementary material for: Use of Simulated Patients to Evaluate Combined Oral Contraceptive Dispensing Practices of Community Pharmacists
Source: PLoS One. 2013 Dec 4;8(12):e79875. doi: 10.1371/journal.pone.0079875 (PMC3853625; doi:10.1371/journal.pone.0079875)
Supplement: Appendix S1 — Sample data collection sheet. (DOC) [file pone.0079875.s001.doc]

**Appendix 1-** Sample data collection sheet

Simulated patient name:

Community pharmacy address:

Date: Time started: Time finished: Duration time:

Screen for safe use

*Did the community pharmacist asked the following questions*:

Date of last delivery? □ yes □ no

Are you currently breastfeeding? Date of last delivery? □ yes □ no

How old are you? □ yes □ no

Do you smoke? □ yes □ no

How many cigarettes do you smoke per day? □ yes □ no

Do you have high blood pressure? Do you take medicine for high blood pressure? □ yes □ no

Do you currently have gall bladder disease? □ yes □ no

Are you currently using medication for high cholesterol? □ yes □ no

Do you have diabetes? If yes, do you have problems with your kidneys, eyes, or nerves related to diabetes? Have you been told that you have problems with your arteries or veins related to your diabetes? Have you had diabetes for more than 20 years? □ yes □ no

Do you have seizures (epilepsy) or tuberculosis? If so, are you using any of the following medications: rifampicin, phenytoin, carbamazepine, barbiturates, primidone, topiramate, oxcarbazepine? □ yes □ no

Do you have migraine headaches? If so, when you get these headaches do you have any of these associated symptoms: numbness, weakness, difficulty seeing, nausea or vomiting, or sensitivity to light? Are your headaches usually on one side or both sides? When you get one of these headaches, does it interfere with your normal activities a little, somewhat, or a lot? □ yes □ no

Have you had a blood clot in your lung or in your leg (not just varicose veins)? □ yes □ no

Do you have a major surgery recently? □ yes □ no

Have you had a heart attack or stroke? □ yes □ no

Do you have heart disease? If so, what kind? □ yes □ no

Do you currently have breast cancer or have you had breast cancer in the past? □ yes □ no

Do you currently have liver disease (like hepatitis or cirrhosis) or have you had liver cancer in the past? □ yes □ no

*Number of closed-ended questions asked*:

*Number of open-ended questions asked*:

*Write the questions made by the community pharmacists (copy exactly the question)*:

1- _______________________________________________________________

2- _______________________________________________________________

3- _______________________________________________________________

4- _______________________________________________________________

5- _______________________________________________________________

Did the community pharmacist measured patients´ blood pressure level? □ yes □ no

*Global evaluation of screen for safe use of COC*:

□ Very poor □ Poor □ Moderate □ Good □ Very good

Counseling for correct use:

*Did the community pharmacist counsel regarding*

The name of the COC? □ yes □ no

The description of the COC? □ yes □ no

The indication of the COC? □ yes □ no

The route of administration of the COC? □ yes □ no

The dose of the COC? □ yes □ no

The dosage form of the COC? □ yes □ no

The directions for use of the COC? □ yes □ no

The duration of therapy of the COC? □ yes □ no

The special directions of the COC? □ yes □ no

The precautions of the COC use? □ yes □ no

The adverse effects of the COC? □ yes □ no

What to do in the occurrence of adverse effects of the COC? □ yes □ no

The contraindications of the COC? □ yes □ no

*Write the counseling gave by the community pharmacists (copy exactly the question):*

1- _______________________________________________________________

2- _______________________________________________________________

3- _______________________________________________________________

4- _______________________________________________________________

5- _______________________________________________________________

*Global evaluation of counsel for correct use of COC:*

□ Very poor □ Poor □ Moderate □ Good □ Very good
